# Detection of Atrial Fibrillation Episodes based on 3D Algebraic Relationships between Cardiac Intervals

**DOI:** 10.3390/diagnostics12122919

**Published:** 2022-11-23

**Authors:** Naseha Wafa Qammar, Vaiva Šiaučiūnaitė, Vytautas Zabiela, Alfonsas Vainoras, Minvydas Ragulskis

**Affiliations:** 1 Department of Mathematical Modelling, Kaunas University of Technology, LT-51368 Kaunas, Lithuania; 2 Cardiology Institute, The Lithuanian University of Health Sciences, Mickeviciaus g.9, LT-44307 Kaunas, Lithuania

**Keywords:** atrial fibrillation, perfect matrix of Lagrange differences, statistical indicator, decision support system

## Abstract

In this study, the notion of perfect matrices of Lagrange differences is employed to detect atrial fibrillation episodes based on three ECG parameters (JT interval, QRS interval, RR interval). The case study comprised 8 healthy individuals and 7 unhealthy individuals, and the mean and standard deviation of age was 65.84 ± 1.4 years, height was 1.75 ± 0.12 m, and weight was 79.4 ± 0.9 kg. Initially, it was demonstrated that the sensitivity of algebraic relationships between cardiac intervals increases when the dimension of the perfect matrices of Lagrange differences is extended from two to three. The baseline dataset was established using statistical algorithms for classification by means of the developed decision support system. The classification helps to determine whether the new incoming candidate has indications of atrial fibrillation or not. The application of probability distribution graphs and semi-gauge indicator techniques aided in visualizing the categorization of the new candidates. Though the study’s data are limited, this work provides a strong foundation for (1) validating the sensitivity of the perfect matrices of Lagrange differences, (2) establishing a robust baseline dataset for supervised classification, and (3) classifying new incoming candidates within the classification framework. From a clinical standpoint, the developed approach assists in the early detection of atrial fibrillation in an individual.

## 1. Introduction

### 1.1. Existing Diagnostics Techniques for Atrial Fibrillation

Atrial fibrillation (AF) is linked to an increased risk of cardiovascular events such as cardiovascular mortality, major cardiovascular events, heart failure, ischemic heart disease, sudden cardiac death, and stroke [1,2]. Numerous symptoms, such as fatigue, palpitations, shortness of breath, and chest pain, are experienced by AF patients. Some patients have no symptoms, which is known as asymptomatic or “silent” AF. Asymptomatic AF has serious clinical consequences. Patients with undiagnosed AF may develop life-threatening thromboembolic complications or tachycardia-mediated cardiomyopathy [3,4]. People with AF disorders often have no symptoms or vague signs because AF is frequently intermittent. This combination consequently makes detection and diagnosis challenging. Most of the time, AF is not noticeable until a person experiences a serious health issue and seeks therapy [2]. One of the key tactics for enhancing AF identification and possibly lowering AF-related stroke, mortality, and healthcare expenditures is screening for AF [4]. The rhythm disturbance’s frequent silence makes it difficult to detect AF early, which is a serious concern. The so-called asymptomatic AF is unknown to patients in around one-third of those who have this arrhythmia. Much earlier detection of the arrhythmia may enable the prompt introduction of medicines to protect patients not only from the consequences of the arrhythmia, but also from the progression of AF from a treatable illness into an untreatable issue [5].

There are various heart health monitoring tools available on the market, such as AliveCor [2], that show promise, particularly for patients with intermittent AF. However, this equipment is expensive and unable to identify those with AF condition symptoms. Today, a variety of methods, including Single Lead, Holter Monitoring, Mobile Telemetry Monitoring, and Implantable Loop Recorders, are available for use in AF detection outside of the hospital setting. Spot single-lead ECG monitors, such as the AliveCor, need the patient to identify symptoms, document them, and discuss them with their clinician [2]. It can be concluded that current heart health monitoring methods are either excessive or call for specialized, pricey equipment that must be used in conjunction with substantial signal processing to produce better quality findings. People with known or potential cardiovascular problems must have access to a low-cost heart health monitoring system in order to lower the expense of medical examinations, lessen their worry, and receive timely care.

The electrocardiogram (ECG) visual examination is the gold standard for identifying AF. An ECG is required to identify AF; nevertheless, an erratic pulse can increase the suspicion that it may be present [6,7]. A 12-lead ECG can be used to confirm the presence of AF, a frequent persistent arrhythmia that is frequently asymptomatic [2]. In addition to the severe irregularity of the RR interval, P waves vanish and are replaced by irregular fibrillation waves (f waves) of various sizes and forms [8,9].

### 1.2. Perfect Matrices of Lagrange Differences as a Method for ECG Signal Analysis

ECG signals have been examined over time to detect and examine various cardiovascular diseases [9]. Before delving into the analysis techniques of the ECG signal, it is important to recall that the ECG signal is a complex signal in its behavior [10,11]. The complexity explicates how the various ECG parameters [12] have significant variation between them, which is caused by various physiological and pathological factors, so therefore, no definite mathematical model can characterize the relationship between ECG parameters, even for a specific individual [11,13].

There is always an attempt to analyze the ECG signal in such a way that the data with the vast bulk of the extraction of clinically relevant features are available, which contain all the important information of the original ECG signal and thus operate as the signal’s substitute for further analysis. Several computational approaches including deep learning methods, such as feature extraction and dimensionality reduction, are well known for conducting the analysis of ECG. These techniques are helpful for increasing clinical research by gaining a better understanding of medical challenges [14,15]. Feature extraction technique can occur in the frequency domain, time domain, or frequency–time domain analysis [16]. Noujaim et al. [17,18,19] propose that the behavior of the cardiovascular system is “fractal-like”, attempting to retain adaptive variability demonstrating non-linearity in the cardiovascular system rather than stability. As a result, it makes more sense to seek a solution to dynamic processes that are complicated in nature [11]. A range of well-known nonlinear analytic techniques for analyzing ECG data have been used due to their robustness. For instance, reconstructed phase space analysis [20], Lyapunov exponents [21,22], correlation dimension [23,24,25], detrended fluctuation analysis (DFA) [26], recurrence plot and Poincaré plots [27], and possibly additional nonlinear analytic approaches for ECG analysis are becoming increasingly popular.

It is essential to surpass the boundaries of conventional nonlinear computation techniques to determine potentially accurate, faster, and more reliable solutions for the analysis of the ECG parameter. The idea of the perfect matrices of Lagrange differences has been proposed and proved for the different nonlinear time series analysis applications in several studies [10,11,28]. One of the objectives of this paper is to show that the expansion of the matrix dimension does result in the increased sensitivity of the algebraic relationships between cardiac intervals, which provides a more accurate platform for the early detection of atrial fibrillation. First, the architecture of the third-order perfect matrices of Lagrange differences is introduced. Then, the sensitivity of the proposed architecture is demonstrated by comparing the proposed architecture to the second-order perfect matrices of Lagrange differences used in previous studies. The rest of the structure of the paper is as follows. The notion of the perfect matrices of Lagrange differences is explained (this part is basically a recall from the work [10,11] and is hence named “*Preliminary Synopsis*”). The perfect matrices of Lagrange differences are used to explain how to interpret the relationships between the three cardiac intervals (JT interval, QRS interval, RR interval). It is demonstrated (and validated) that the expansion of the dimension of the matrix serves as an accurate platform for assessing the ECG signal. Subsequently, using the one sigma rule, statistical operations are performed on the dataset to establish an observation window for the classification purposes. In addition to the statistical analysis, it is shown how the generated probability index and the semi-gauge representation help to serve as the decision support instrument for the detection of early AF episodes.

## 2. Methods

### 2.1. The Description of the Experimental Setup

The three cardiac intervals, (i) JT interval, (ii) QRS interval, and (iii) RR interval, are registered as three time series, as shown in Figure 2. The ECG signal is recorded under the “No Load” condition, which indicates that the participants did not exercise or perform any stress tests while the ECG recordings were registered.

### 2.2. Participants

Participants in this study were divided into two groups: healthy and unhealthy individuals. It is worth mentioning that the ECG data collected for both groups showed a proclivity for atrial fibrillation (AF). Antiarrhythmic medication was not reported to be used by either group of subjects; therefore, the cohorts were divided and categorized as healthy or unhealthy. The cohort of healthy individuals comprised 8 people, whereas the cohort of unhealthy individuals comprised 7 people. The mean and standard deviation of age was 65.84 ± 1.4 years; of height, 1.75 ± 0.12 m; and of weight, 79.4 ± 0.9 kg. For purposes of confidentiality, the candidates’ names were labeled as H1–H8, which indicate healthy candidates, and UH1-UH7, which denote unhealthy candidates (see Tables 3–6).

### 2.3. Ethics Statement

The research met all applicable standards for the ethics of experimentation in accordance with the Declaration of Helsinki as reflected in prior approval by the Regional Biomedical Research Ethics Committee of the Lithuanian University of Health Sciences (ID No. BE-2-4, 17 March 2016). The permit to perform biomedical investigation was granted by the LUHS Bioethics Committee (see the Institutional Review Board Statement). Participants provided written informed consent prior to the experiment.

### 2.4. The Description of the Proposed Algorithm

#### 2.4.1. Preliminary Synopsis

Let us begin by presenting a brief overview of the three cardiac intervals examined in this study (JT interval, QRS interval, and RR interval). Figure 2 depicts a time series of the three cardiac intervals for one of the candidates. In Figure 2, the x-axis represents the time in minutes, whereas the y-axis represents the time series recorded for the each of the three cardiac intervals. Let us denote each of these three time series as $x=\left(x_{1},x_{2},x_{3},\cdots \cdots ,x_{i}\right)$, $y=\left(y_{1},y_{2},y_{3},\cdots \cdots ,y_{i}\right)$, and $z=\left(z_{1},z_{2},z_{3},\cdots \cdots ,z_{i}\right)$, where i is the total number of heartbeats recorded throughout the experiment.

The key concept, notion, and underlying conditions for the generation of 2-by-2 perfect matrices of Lagrange differences and the corresponding algebraic relationship have been previously thoroughly explained and verified [10,11,28]. Furthermore, the fact that derivatives tend to amplify noise, as well as the implications of reasonable mathematical manipulations for the extraction of the most relevant information from the ECG signal, have already been addressed in [10,11,28] and will not be explained in this work. What makes this research distinct from previous approaches is that (1) the order of the matrices is increased, thus enhancing the sensitivity of the proposed algorithm, and (2) the idea of employing this method for early detection of AF from a cohort of healthy and unhealthy individuals. The next section discusses the architecture of the 3-by-3 perfect matrices of Lagrange differences and then reflects on its potential sensitivity.

#### 2.4.2. The Architecture of Third-Order Square Matrices of Lagrange Differences

The definition of 2-by-2 perfect matrices of Lagrange differences is given in [10,11,28]. Each element of the matrix can be either a zeroth-order difference or a first-order difference. The following conditions must hold true for the matrix in order be a perfect matrix of Lagrange differences [10,11,28]:

1. All elements of the matrix must be different.
2. Zeroth-order differences are located on the main diagonal.
3. First-order differences are located on the secondary diagonal.
4. The matrix is balanced with respect to time (the sum of all time lags is equal to zero).
5. The matrix is balanced with respect to lexicographic variables (the number of different symbols must be the same).

Let us assume that index n denotes the current moment and δ represents the time lag (δϵℕ). Then, the structure of the 2-by-2 perfect matrix of Lagrange differences reads [10,11,28]:

(1) $$\left[\begin{array}{cc} x_{n} & x_{n+\delta }-y_{n+\delta }\\ x_{n-\delta }-y_{n-\delta } & y_{n} \end{array}\right]$$

**Figure 1:**
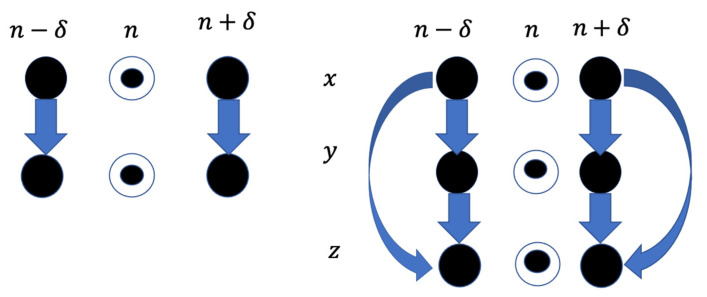
A schematic diagram illustrating the architecture of 2-by-2 and 3-by-3 perfect matrices of Lagrange differences. Zeroth-order differences (the diagonal elements of the matrices) are depicted in circles. First-order differences are depicted by arrows connecting respective elements.

The schematic representation of this 2-by-2 perfect matrix of Lagrange differences can be illustrated by the diagram in Figure 1. The diagonal elements of the matrix are shown in circles. The first-order Lagrange differences are depicted by arrows connecting respective elements.

**Figure 2:**
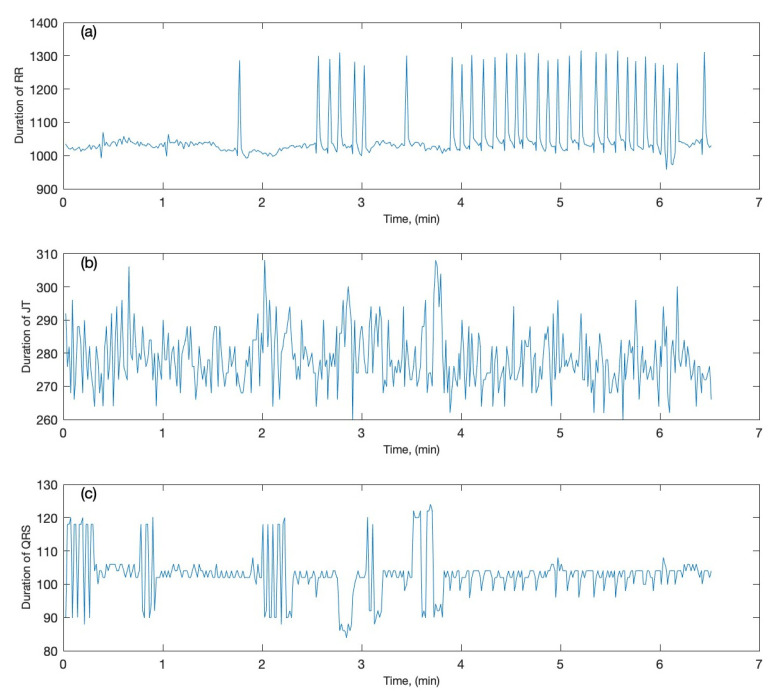
The three recorded cardiac intervals are plotted as time series (the time is in minutes): (**a**) the RR interval; (**b**) the JT interval; (**c**) the QRS interval.

Let us consider the three different time series x*,* y*,* and z (Table 1) also shown in Figure 2. The architecture of the 3-by-3 matrix can be naturally expanded by scaling the structure of Equation (2).

(2) $$\left[\begin{array}{ccc} x_{n} & y_{n+\delta }-x_{n+\delta } & z_{n+\delta }-x_{n+\delta }\\ y_{n-\delta }-x_{n-\delta } & y_{n} & z_{n+\delta }-y_{n+\delta }\\ z_{n-\delta }-x_{n-\delta } & z_{n-\delta }-y_{n-\delta } & z_{n} \end{array}\right]$$

It can be observed that all requirements for perfect matrices of Lagrange differences hold true for Equation (2). Therefore, the matrix defined by Equation (2) is also a perfect matrix of Lagrange differences. The schematic representation of the 3-by-3 perfect matrix of Lagrange differences is depicted in Figure 1. Again, the diagonal elements (the zeroth-order Lagrange differences) are shown in circles; all first-order Lagrange differences are depicted by arrows connecting respective elements.

The algebraic relationship between two cardiac intervals (sequences x and y) is defined by a mapping function $ℱ:{\mathbb{R}}^{\left(2*2\right)}\to {\mathbb{R}}^{1}$, which transforms each perfect matrix of Lagrange differences to a scalar variable. Different mapping functions are used in [10,11,28] (the discriminant of the matrix, the modulus of the maximal eigenvalue of the matrix, the norm of the matrix). The same mapping function $ℱ:{\mathbb{R}}^{\left(3*3\right)}\to {\mathbb{R}}^{1}$ should be defined for the 3-by-3 perfect matrix of Lagrange differences. The norm of the matrix is set at the mapping function in all further computations. Internal and external smoothing of the mapped scalar algebraic relationship between cardiac intervals are executed after the mapping procedure [28]; the same radiuses of internal and external smoothing are adapted from [28].

#### 2.4.3. The Sensitivity of the Proposed Algorithm

**Figure 3:**
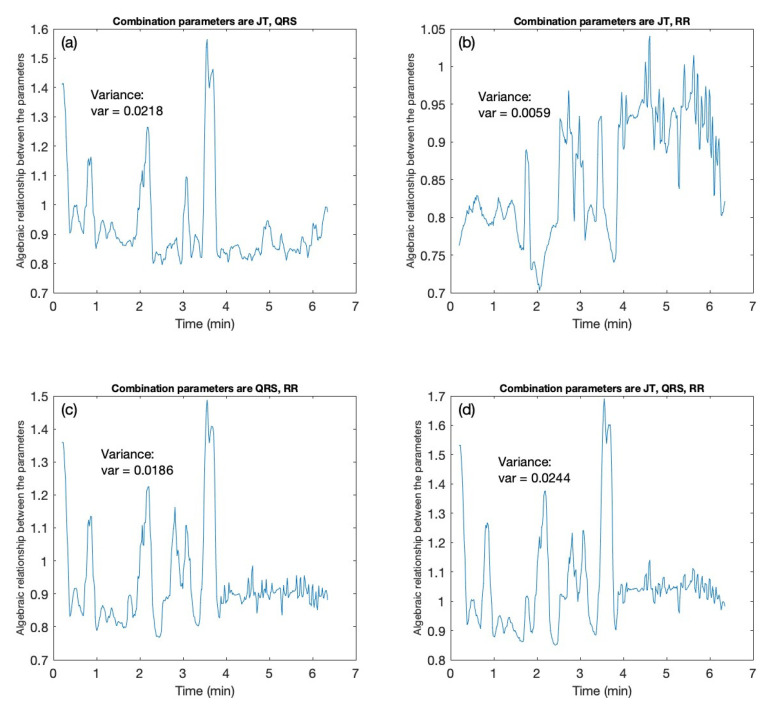
The sensitivity of 3-by-3 matrices vs 2-by-2 matrices is demonstrated for one of the candidates. The *x*-axis denotes the time in minutes. The *y*-axis demonstrates the algebraic relationship for the 2-by-2 (**a**–**c**) and 3-by-3 (**d**) matrix combination for the cardiac intervals JT, QRS, RR. (**a**) The 2-by-2 matrix algebraic relation is computed for the combination of cardiac intervals JT, QRS. (**b**) The 2-by-2 matrix algebraic relation is computed for the combination of cardiac intervals JT, RR. (**c**) The 2-by-2 matrix algebraic relationship is computed for the cardiac intervals QRS, RR. (**d**) The 3-by-3 matrix algebraic relationship is computed for the combination of cardiac intervals JT, QRS, RR.

After illustrating the structure of the 3-by-3 perfect matrices of Lagrange differences, the sensitivity of the proposed architecture is explored. To this end, one of the candidates from the cohort of individuals is employed as an example. The ECG signal is recorded for the candidate (Figure 2). Initially, all computations are performed with 2-by-2 perfect matrices of Lagrange differences, as described in Section 2. The algebraic relationship between JT and QRS intervals is shown in Figure 3a; the algebraic relationship between JT and RR intervals is shown in Figure 3b; the algebraic relationship between QRS and RR intervals is shown in Figure 3c.

These computations are repeated with all recorded cardiac intervals (JT, QRS, RR intervals). Three scalar time series are mapped into a sequence of 3-by-3 matrices, as shown in Figure 1. It is to be noted that the order of the parameter also has an important role in defining the sensitivity of the proposed algorithm. The order of the parameters in this study is fixed with JT interval as x, QRS interval as y, and RR interval as z (see Section 3 for more details). Once the three parameters are according to the proposed architecture, they are evaluated by using computational techniques discussed above. (1) The recorded time series are mapped into the trajectory matrices. (2) With the mapping (ℱ) technique, the trajectory of the matrices is transformed into a scalar time series ($ℱ:{\mathbb{R}}^{\left(3*3\right)}\to {\mathbb{R}}^{1})$. As noted previously, the mapping function ℱ is defined as the norm of the 3-by-3 perfect matrix of Lagrange differences. Finally, the internal and external smoothing techniques are applied to smooth the mapped scalar sequence.

The sensitivity of the algebraic relationship revealed by the 2-by-2 and 3-by-3 perfect matrices of Lagrange differences is measured by the variability (the variance) of the scalar mapped signal depicted in Figure 3. The algebraic relationship between JT and QRS intervals yields the variance value of 0.0218 (Figure 3a). Analogously, the JT–RR relationship yields 0.0059 (Figure 3b); the QRS–RR relationship yields 0.0186 (Figure 3c). The 3-by-3 algebraic relationship between JT, QRS, and RR intervals yields the variance value of 0.0244 (Figure 3d). This increase in the sensitivity of the algebraic relationship can be explained by the following reasoning. Only two of four terms represented differences in the structure of a 2-by-2 perfect matrix of Lagrange differences. However, six of nine terms represented differences in the structure of a 3-by-3 perfect matrix of Lagrange differences. As mentioned previously, the differences better reflect the changes in the inter-connected system represented by related synchronized cardiac intervals representing every consecutive heart contraction. Moreover, the information fed into the algorithm is much larger (three cardiac intervals instead of two). Therefore, a higher sensitivity of the 3-by-3 matrix architecture is not surprising.

The values of the variances obtained from the second- and third-order perfect matrices of Lagrange differences are tabulated in Table 2. With the comparison between the variance values for both second- and third-order matrices, the sensitivity of the proposed algorithm is highly exhibited by the 3-by-3 perfect matrices of Lagrange differences. The results tabulated in Table 2 and shown in Figure 3 confirm the hypothesis of this study. Before we delve into the results and discussions, we review the number of computations performed on our dataset to determine (1) which combination patterns exhibit more sensitivity from the 3-by-3 perfect matrices of Lagrange differences and (2) which combination patterns produced the most likely statistical outcomes for the statistical computations.

#### 2.4.4. Trials Computed upon the Dataset with Different Combination Patterns

This section explores different combinations of the parameters to determine which combination is more sensitive in characterizing the algebraic relationship between the parameters and which statistical parameters should be chosen for the subsequent computations. With the number of vectors as three, k=3, the possible number of combinations that could be made is $2^{k}=2^{3}=8$. The computations are performed on all the possible combinations (i.e., $2^{k}=2^{3}$); a few of them are highlighted in Table 3, Table 4, Table 5 and Table 6. As mentioned before, the computations were conducted with two main objectives: (1) by altering the parameters’ order, it is intended to assess the sensitivity of the proposed algorithm, and (2) to look for the most suitable statistical analysis parameter for further computations, whether it is variance ($\sigma^{2}$), mean (σ), median (x~), or standard deviation ($\sigma_{x}$). In Table 3, the computational techniques are performed for the combination parameters (QRS interval, RR interval, and JT interval). First, the variance ($\sigma^{2}$), mean (σ), median (x~), and standard deviation ($\sigma_{x}$) are computed for the sequence representing the algebraic relationship between cardiac intervals (see Table 3). To obtain more statistically significant results, the one sigma rule is applied to the smoothed sequence for the cohort of both healthy and unhealthy people. Note that the one sigma rule approach applied here must not be confused with the one sigma rule used subsequently in the study for the variance data. At this point in the study, the goal of employing the one sigma rule is to identify the variability in the algebraic relationship between the various combinations of parameters. In addition, the one sigma rule is applied to the median and standard deviation of the smoothed algebraic sequence, but later in the study, the one sigma rule will be applied to the variance data when the statistical manipulations and computer-generated procedures are applied to it.

As the implication of the one sigma rule, the upper boundary ($x\sim_{H}+\sigma_{H}$) and lower boundary ($x\sim_{H}-\sigma_{H}$) are defined. Subsequently, the areas (A) above and below the boundaries are computed. With the boundaries defined, statistical conditions are applied to the smoothed sequence. If the smooth sequence is greater than the upper limit, then the area above the upper limit is computed, and if the smoothed sequence is less than the lower limit, the area below the lower limit is computed. The final outcome of the overall area computed is denoted by A (see Table 3, Table 4 and Table 5). It makes sense to conduct computational trials to determine the extent of the variability that exists while defining the algebraic relationship between the three parameters. For the combination (QRS interval, RR interval, JT interval), we do see the variability, as shown by the statistical outcomes tabulated in Table 3. The same statistical techniques have been performed for the other three sequences (RR interval, JT interval, and QRS interval; JT interval, RR interval, and QRS interval; JT interval, QRS interval, and RR interval—see Table 4, Table 5 and Table 6). It is worth noting that while variability exists in all of these combinations, upon close inspection of the statistical data, it is particularly significant for the parameter combination of JT interval, QRS interval, and RR interval (see Table 3, Table 4, Table 5 and Table 6).

Furthermore, upon the comprehensive review of the mean (σ), median (x~), and standard deviation ($\sigma_{x}$) data for the cohort of healthy and unhealthy individuals, it is revealed that the variance ($\sigma^{2}$) data are considerably distinctive and sensitive in their conclusions for both cohorts in all combinations (see Table 3, Table 4, Table 5 and Table 6) and can be utilized to generate a reliable baseline dataset for the classification, which can then be used to classify new incoming candidates. Therefore, the variance ($\sigma^{2}$) data are chosen for generating the Gaussian distribution graphs and producing the baseline dataset. In the next section, the discussion on the statistical analysis techniques and the subsequent outcomes are elaborated in detail.

#### 2.4.5. The Development of the Decision Support System

**Figure 4:**
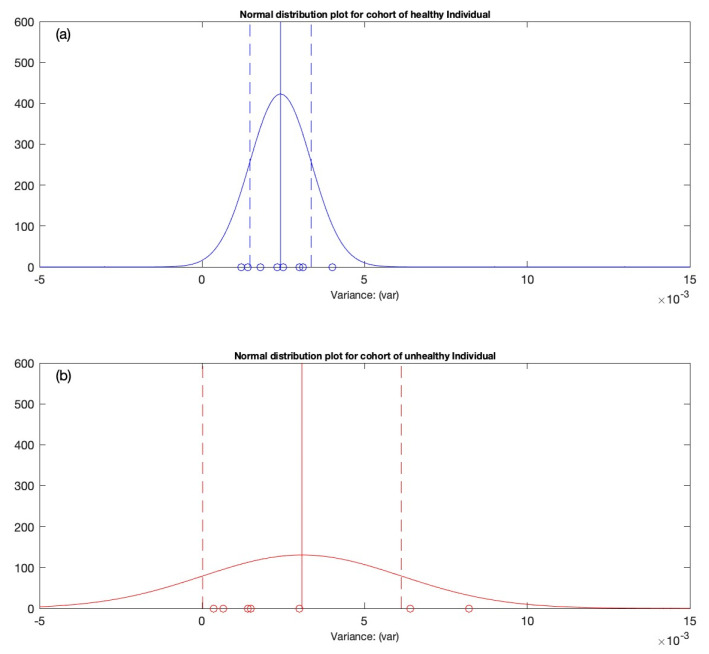
The distribution of individuals in each cohort is approximated by the Gaussian distribut-ion. (**a**) The Gaussian distribution plot computed for the cohort of healthy individuals. (**b**) The Gaussian distribution plot computed for the cohort of unhealthy individuals.

**Figure 5:**
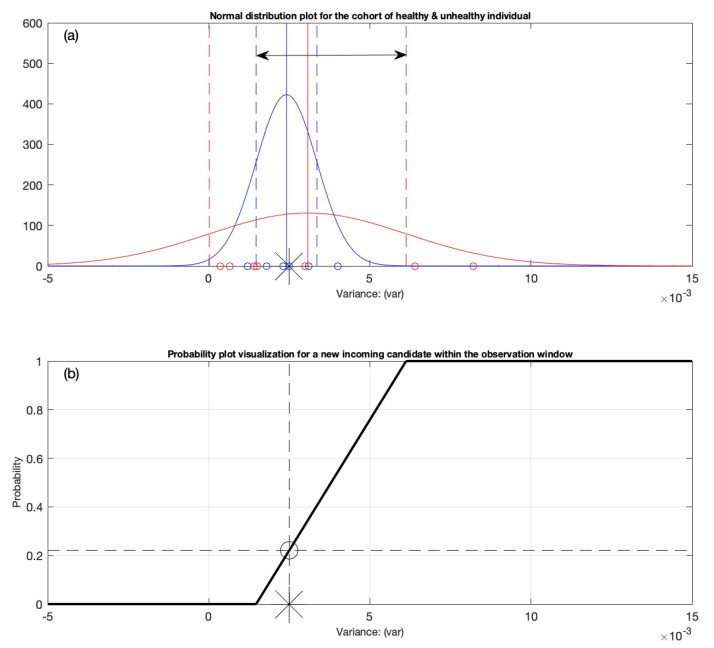
(**a**) The construction of the variation interval between the mean of the healthy distribution minus the standard deviation of the healthy distribution, and the mean of the unhealthy distribution plus the standard deviation of the unhealthy distribution. The variation interval is indicated by a double arrow and is used for the classification of a new incoming candidate. (**b**) The decision support system is depicted as a probability distribution plot with a candidate’s likelihood (0.22) of having AF, indicated by an asterisk sign.

Following the sensitivity assessment of the 3-by-3 matrices based on the variance values (highlighted and underlined in Table 6), the statistical computations are performed for the cohort of healthy and unhealthy individuals. The retrieved knowledge from the algebraic relationship is employed to conduct the statistical analysis. For example, the variance values are used to perform the statistical analysis for further computations. The variance values will be employed to estimate the Gaussian distribution plot for both cohorts (see Figure 4). The distribution will help to determine how evenly the data are distributed along the x-axis. Since the distribution graph will assist in establishing the foundations for classification, the one sigma rule is applied to the Gaussian distribution plot for classification (see Figure 5a). The data fitted to the classification serve as the foundation for classifying the new incoming individual in order to determine whether he/she falls into the category of a healthy or unhealthy individual based on the classification rules established in Table 7. To strengthen the visualization of classification of the new candidate, a graphical representation of the probability distribution graph is proposed, followed by a gauge representation with coloring indication for generating the warning system. If an incoming person within the classification demonstrates either healthy or unhealthy behavior, the classification approach will single out the individual according to his/her ECG registering, and the warning gauge will provide a graphical depiction via a color on the gauge that might be green, yellow, or red (based on the individual’s behavior). For example, if a person enters the classification and exhibits the behavior of a healthy person, the gauge arrow will point towards the green color, indicating that the incoming person is healthy. On the other hand, if the incoming person indicates that he/she falls even slightly towards the unhealthy category, the indication gauge arrow will point towards yellow, which means that the incoming person may have indications of atrial fibrillation based on his/her ECG registering. Finally, if the approaching individual falls far towards the right of the distribution graph, it strongly indicates that the incoming person has a higher risk of having atrial fibrillation episodes and will be indicated by the color red. Each of these scenarios are discussed more extensively in Section 3.

## 3. Results and Discussion

First, the sensitivity of the proposed architecture is analyzed to assess the three ECG parameters (JT interval, QRS interval, RR interval). As said earlier, the dataset comprises a cohort of eight healthy individuals and seven unhealthy individuals. For each candidate, the ECG signal is presented as a time series and is characterized as scalar vectors; x, y, and z. According to the architecture shown in Figure 1 and tabulated in Table 1, each of these scalar vectors is passed through the routine of perfect matrices of Lagrange differences. In other words, the three scalar vectors are transferred onto a trajectory of matrices. Figure 3 depicts the algebraic relationship between the vectors x, y, and z for one of the candidates, but the computations were performed for the entire cohort of 15 people (see Table 6).

The matrix trajectory for each individual in the cohort is translated into a scalar time series using the norm of the matrix, where mapping is represented as $ℱ:{\mathbb{R}}^{\left(3*3\right)}\to {\mathbb{R}}^{1}$. Finally, the signal is smoothed using internal and external smoothing techniques. The entire computation resulted in the final smooth singular scalar vector, which contains most of the original signal’s information. Table 6 illustrates the outcomes of the matrix’s sensitivity analysis (as mentioned in Section 2.4.3, our attention is entirely focused on the variance data for both cohorts). Up to this point, mathematical computations are performed for each individual in the cohort in order to determine the algebraic relationship between the JT, QRS, and RR intervals according to the architecture proposed earlier. In the next section, the statistical computational techniques are discussed in detail.

### 3.1. Performing the Statistical Analysis

The statistical analysis is performed on the variance dataset presented Table 6. To begin with, the Anderson–Darling goodness-of-fit hypothesis test is conducted to determine whether the data values for the healthy and unhealthy individuals are of a normal distribution. Once the Anderson–Darling goodness-of-fit hypothesis test confirms the normal distribution of the dataset, the estimates of the mean (mu), standard deviation (sigma), and 95% confidence intervals are determined using the computer-generated built-in algorithms. Finally, the distribution graphs are created using the machine-provided probability density function. In Figure 4, the x-axis represents the distributed data (which are variance values produced from the proposed matrix architecture) for the cohort of healthy and unhealthy individual, whereas the y-axis shows the probability density of the dataset. To distinguish between the healthy and unhealthy cohorts, different color schemes are employed, such as blue for healthy individuals and red for unhealthy people. In the next section, the criterion for generating the observation interval is employed, which is used to classify the new incoming individual.

### 3.2. Generation of the Variation Interval

Figure 4 indicates that the distribution has an optimal fit for the cohort of healthy and unhealthy individuals. The observation interval is defined for classification purposes. The one sigma rule is applied on the normally distributed data shown in Figure 4. For this, the variation interval’s boundaries must first be defined. The boundaries are indicated as right $\mu_{\mathrm{uh}}+\sigma_{\mathrm{uh}}$ and left $\mu_{h}-\sigma_{h}$ ends of the interval. This is indicated in Figure 5a by a double arrow linking the two boundaries. The new entrants into the observation window will be classified using this variation interval. After defining the observation interval, it is essential to build the probability distribution graphical representation and develop the semi-gauge indication tool. A set of statistical conditions is required for this purpose, as shown in Table 7. Before defining the conditions, a few variables must be defined. For example, the new candidate is denoted by C, and the variable IND (indicator) is introduced to represent the new person’s location within the variation interval. The indices “h” and “uh” denote healthy individuals and unhealthy individuals, respectively. The right side of the variation interval is denoted as $\mu_{\mathrm{uh}}+\sigma_{\mathrm{uh}}$ and the left end is denoted as $\mu_{h}-\sigma_{h}$. The location of the new arriving candidate may be identified by the asterisk sign located along the x-axis depending on its ECG registration (see Figure 5). The conditions defined for the classification of the new incoming candidate are discussed below.

#### 3.2.1. Condition 1

If the incoming candidate’s ECG registering value is less than or equal to the left side of the variation interval, which is denoted as C $\leq \;\mu_{h}-\sigma_{h}$ (see Table 7), the indicator (IND) will be zero, and the person will fall towards the left side of the variation interval, indicating that he/she is a healthy candidate.

#### 3.2.2. Condition 2

If the incoming candidate’s ECG registering value is larger than or equal to the right side of the variation interval, which is referred to as $C\geq {\;\mu }_{\mathrm{uh}}+\sigma_{uh}$ (see Table 7), then the person will fall towards the right side of the variation interval, indicating that he/she is a unhealthy candidate.

#### 3.2.3. Condition 3

If the incoming candidate’s value falls between the variation interval, which is codified as $u_{h}-\sigma_{h}\leq C\leq \mu_{uh}+\sigma_{uh}$ (see Table 7), the arriving candidate C falls in between the variation interval.

Because the effect of atrial fibrillation is not expressed during ECG registration, the distribution function is wide and the standard deviation is high. This implies that the subjects may have experienced asymptomatic atrial fibrillation. There could have been some “silent” AF candidates even among the healthy people. As a result, we created an algorithm that could aid in their classification.

**Figure 6:**
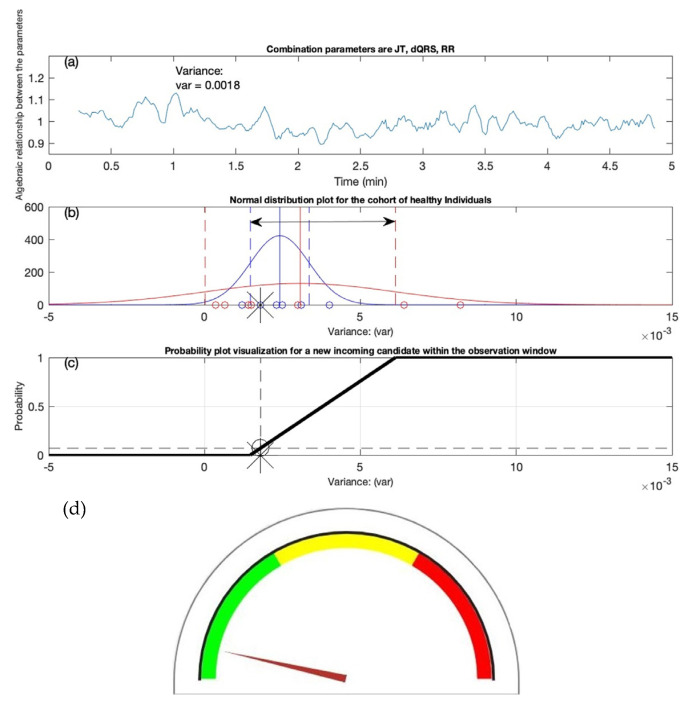
Test candidate #1. (**a**) The algebraic relation is shown for the combination of JT, RR, and QRS intervals. (**b**) The classification of the candidate is marked with an asterisk indicator. (**c**) The decision support system recommends the probability of the AF equal to 0.07. (**d**) The semi-gauge representation is exhibited for the candidate with the arrow of the gauge pointing towards green, indicating that the person is classified as a healthy individual.

**Figure 7:**
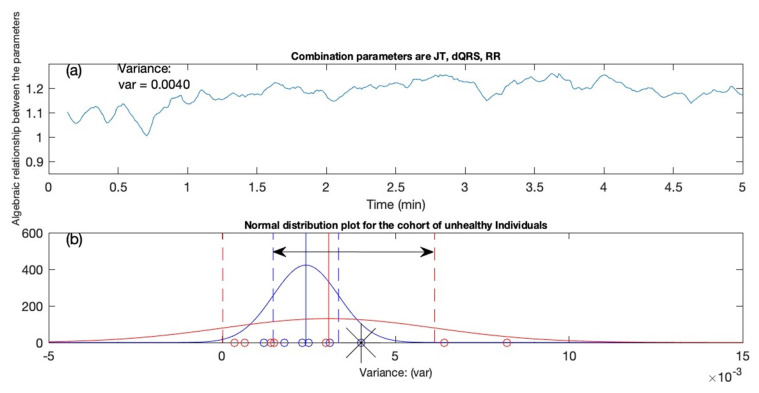

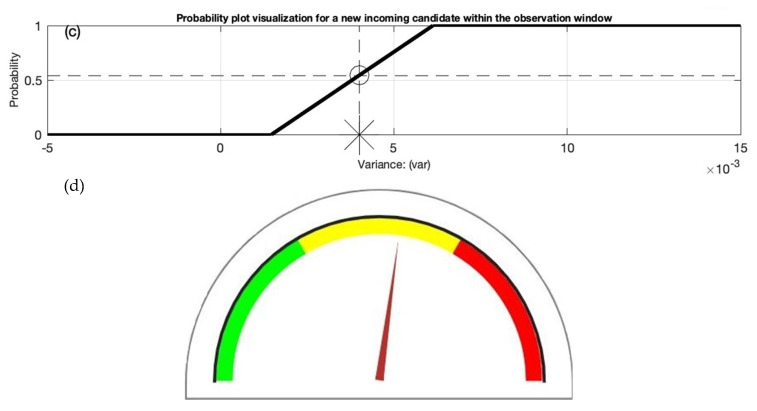
Test candidate #2. (**a**) The algebraic relation is shown for the combination of JT, QRS, and RR intervals. (**b**) The classification of the candidate is marked with an asterisk indicator. (**c**) The decision support system recommends the probability of the AF equal to 0.54. (**d**) The semi-gauge representation is exhibited for the candidate with the arrow of the gauge pointing towards yellow, indicating that the person is classified as an unhealthy individual.

To supplement the visual representation, we incorporate a semi-gauge indication tool for designing a warning system. This is accomplished by adopting a color scheme that is solely dependent on the conditions specified in Table 7. Depending on the ECG registering of the new candidate within the variation interval, the gauge arrow will point towards either green, yellow, or red (see Figure 6d and Figure 7d). In the next section, the two test applicants are analyzed to determine where their ECG recordings fall after classification within the variation interval.

### 3.3. First Test Candidate

Figure 6 depicts the example of one of the test candidates. In Figure 6a, the x-axis represents the time (minutes) and the y-axis represents the algebraic relationship between the three parameters (JT interval, QRS interval, RR interval). The computational techniques resulted in a variance value of 0.0018, as shown in Figure 6a. The resulting variance value is subjected to the classification conditions described in Table 7, and it is noted that the candidate appears to fall on the left side C $\leq \mu_{h}-\sigma_{h}$ of the variation interval. The location of the test candidate is indicated by the asterisk sign in Figure 6b. Furthermore, when the candidate’s value is passed through the routine of the probability distribution graph, it is found that the candidate had the lowest likelihood (0.07) of having an atrial fibrillation prediction, as this can be seen in Figure 6c. In Figure 6c, the x-axis represents the variance values, and the y-axis represents the probability (0–1). Finally, by using a semi-gauge visualization tool, the color indication scheme is used to indicate where the gauge’s arrow points out. It can be seen that arrow direction is towards the green, indicating that the individual is healthy (see Figure 6d). In the next section, we look at another test candidate’s scenario.

### 3.4. Second Test Candidate

Figure 7 illustrates the example of another test candidate subjected to the proposed techniques described in the previous sections. In Figure 7a, the x-axis represents the time in minutes, and the y-axis shows the algebraic relationship between the parameters (JT interval, QRS interval, and RR interval). The mathematical computations generated a variance value of 0.0040 after passing through the proposed algorithm. When the variance value is compared to the data in Table 7, it is noticed that the test applicant falls into the category of healthy individuals. However, when the variance value is subjected to the classification conditions, it appears that the person is falling towards the extreme right $C\geq {\;\mu }_{\mathrm{uh}}+\sigma_{\mathrm{uh}}$ of the observation interval (see Table 7). The probability distribution graphical representation revealed that the test candidate has a likelihood of 0.54 to have the indication atrial fibrillation, indicated by the asterisk sign (Figure 7c). To demonstrate the severity of the warning, the semi-gauge arrow directed towards the yellow indicates the risk that the individual has an indication of having atrial fibrillation (see Figure 7d).

In the examples of test candidates presented above, it is seen that the candidates initially exhibit the behavior of being a healthy individual, but when their ECG recordings are passed through the entire computation techniques proposed, it is discovered that one of the candidates, despite having a normal ECG, may have indications of atrial fibrillation. As mentioned in the introduction section, some people may not exhibit symptoms of AF, which is known as asymptomatic or “silent” AF and has major clinical repercussions. This was discovered to be the case for our study’s second test candidate.

AF is a pathology, and the identification of an arrhythmia on an ECG is necessary for the diagnosis. Unfortunately, prompt diagnosis of PV might be challenging due to the brief arrhythmic paroxysms and frequent asymptomatic state. To distinguish between paroxysmal and persistent AF in the ECG, signal-averaged parameters were not used when looking for unhealthy and healthy people. Electrocardiography signal-averaged parameters and changes in their relationships between AF patients and healthy people were used to identify significant ECG parameters. Due to the fact that this electrocardiography technology is being used to analyze ECG parameters in patients with AF for the first time, there is no way to compare the study’s findings to those of previous investigations. Bright ECGs were seen throughout the examination, but after more research using this methodology, the trends in the disparities between the parameters and their links suggest that the signal-averaged electrocardiography parameters may be useful for screening in patients with risk factors for stroke, for whom a more thorough examination for asymptomatic AF is appropriate. The surface electrocardiogram (ECG) is the primary tool used for the clinical diagnosis of atrial fibrillation (AF), and AF is distinguished by the absence of a P wave due to the electrical activity being disorganized. The RR interval, which reflects the ventricular interbeat, was proposed as a significant biomarker for AF detection, despite the P wave’s relatively low amplitude and challenging baseline that make its detection difficult [29]. Several publications investigate how the complexity of RR intervals changes as different cardiovascular disorders progress [30,31]. A suitable level of RR interval fluctuations within an organism denotes both healthy function and natural self-regulatory flexibility or resilience. JT interval and myocardial metabolic rate are related (when heart activity is the highest, JT interval is the shortest, and vice versa). The JT range, which is divided into the JTa interval (from point J to the peak of the T wave) and the Te interval, regulates certain electrophysiological events (from the T wave peak to the end of T) [32]. ECG leads with a shorter JT interval, showing earlier repolarization and quicker metabolic changes in particular cardiac areas. The JT interval was found to be the more significant indicator of AF risk than the QT interval, which has been linked to the development of AF. This suggests that ventricular repolarization rather than ventricular depolarization is a better predictor of future AF events (e.g., QRS duration). As previous reports have mainly concentrated on the AF risk associated with the entire QT interval, which includes components of both ventricular depolarization and repolarization, the results of this analysis offer significant insight into the pathophysiology of AF [29]. The interventricular synchronization aspects of the QRS duration parameter may be linked to the intrinsic regulatory system of the heart. The QRS complex, which is a component of the heart’s regulatory system, depicts the spreading of depolarization inside the ventricle and the synchronization of the spreading of depolarization between ventricles. Slower conduction is visible in the heart ventricle in the larger QRS complex. In normal conditions, the duration of the QRS complex can range from 80 to 120 ms. This index is sensitive to changes in sympathetic and parasympathetic nervous system tone. This parameter could be observed shortening in a healthy heart during sympathetic activation, as well as during load. It is prolonged in some heart diseases, such as ischemic heart disease. Patients with prolonged QRS had a higher prevalence of AF, according to the authors of a cross-sectional study involving 25,000 people with left ventricular dysfunction [29]. The majority of methods employed by physicians rely on the analysis of discrete values such as heart rate, blood pressure, or the duration of a single cardiac interval. Comparing ECG features in relation to one another can provide more clinical data, which is oftentimes more insightful than evaluating features alone.

## 4. Limitations

The current research had some limitations. For instance, the study sample was rather modest to start with. In order to evaluate atrial shape and potential connections with P-wave ECG parameters, participants were not subjected to cardiac imaging. Additionally, it makes sense to assess the dynamics of ECG parameter and interface values in addition to their instantaneous values when assessing each subject as a separate complex system.

## 5. Conclusions

In this novel work, it is hypothesized, demonstrated, and proved that increasing the dimensions of the perfect matrices of Lagrange differences from two to three enhances the sensitivity of the proposed architecture and provides sufficient grounds for analyzing the algebraic relationship between the ECG parameters. The idea of perfect matrices of Lagrange differences is exploited to determine the early detection of atrial fibrillation episodes using the ECG parameters JT, QRS, and RR. The computations were carried out on 8 healthy individuals and 7 unhealthy individuals. Despite the modest sample size, the analysis of this study showed solid evidence for defining the algebraic relationship between the parameters. Moreover, several mathematical computational tests were conducted to determine if the mean, median standard deviation, or variance could be employed for classification, and we identified that the variance parameter stood out the most. We decided to choose the variance as a computing tool for generating a baseline dataset. The observation window was then built using the one sigma rule. Furthermore, a set of conditions was also developed based on machine learning techniques to classify the new arriving candidate inside the variation interval. The idea for probability distribution graphs is proposed to indicate whether the new incoming person has the least or greatest likelihood of developing atrial fibrillation symptoms. In addition, a semi-gauge indicator tool was developed in order to construct a warning system based on the classification conditions. We evaluated two test candidates, both of whom initially displayed the behavior of a healthy individual, but when categorized using the classification, it was discovered that one of the test candidates stood out as having an indication of atrial fibrillation.

## 6. Future Work

Since the proposed method is sensitive to describing the algebraic relationship between the ECG parameters, changing the parameters’ order may provide significantly different results. As a result, different ECG parameter combinations are encouraged for further research and experimentation. Nonetheless, this work has the potential to establish a reliable foundation for future research investigating ECG parameters using 3-by-3 perfect matrices of Lagrange differences. As the sample size of the proposed study was modest to begin with, it is encouraged to expand the sample size of the experiment and perform the computations.

## Figures and Tables

**Table 1:** The nine different elements of the time series x,  y, and z with current, time-backward, and time-forward indexes.

$x_{n-\delta }$	$x_{n}$	$x_{n+\delta }$
** $y_{n-\delta }$ **	$y_{n}$	$y_{n+\delta }$
** $z_{n-\delta }$ **	$z_{n}$	$z_{n+\delta }$

**Table 2:** The comparison between the sensitivity of 2-by-2 and 3-by-3 matrices. The variance value for each of the combination is tabulated for one of the candidates (see also Figure 2).

Candidate	Variance Values			
	Combination of JT–QRS Interval	Combination of JT–RR Interval	Combination of QRS–RR Interval	Combination of JT–QRS–RR Interval
	0.0218	0.0059	0.0186	0.0244

**Table 3:** Statistical analysis outcome for the combination of parameters QRS interval, RR interval, and JT interval.

Healthy Candidates (H)							Unhealthy Candidates (UH)						
$\mathrm{Variance}\;\sigma_{H}^{2}$	$\mathrm{Mean}\;\mu_{H}$	$\mathrm{Median}\;x\sim_{H}$	$\mathrm{Standard}\;\mathrm{Deviation}\;\sigma_{H}$	$\mathrm{Upper}\;\mathrm{Limit}\;(x\sim_{H}+\sigma_{H})$	$\mathrm{Lower}\;\mathrm{Limit}\;\left(x\sim_{H}-\sigma_{H}\right)$	$\mathrm{Area}\;A_{H}$	$\mathrm{Variance}\;\sigma_{UH}^{2}$	$\mathrm{Mean}\;\mu_{UH}$	$\mathrm{Median}\;x\sim_{UH}$	$\mathrm{Standard}\;\mathrm{Deviation}\;\sigma_{UH}$	$\mathrm{Upper}\;\mathrm{Limit}\;(x\sim_{UH}+\sigma_{UH})$	$\mathrm{Lower}\;\mathrm{Limit}\;(x\sim_{UH}-\sigma_{UH})$	$\mathrm{Area}\;A_{UH}$
**0.00080583**	0.736	0.734	0.028	0.763	0.707	0.359	**0.0003**	1.168	1.164	0.018	1.182	0.004	0.769
**0.0016751**	0.827	0.821	0.041	0.863	0.781	1.100	**0.0002**	1.281	1.279	0.015	1.294	1.264	0.875
**0.0015197**	0.917	0.916	0.039	0.956	0.878	−0.376	**0.0061**	1.018	1.012	0.078	1.091	0.935	2.062
**0.00073806**	1.031	1.031	0.027	1.059	1.004	0.254	**0.0006**	0.932	0.936	0.025	0.962	0.912	−1.217
**0.0015323**	1.024	1.027	0.039	1.067	0.989	−1.307	**0.0141**	1.422	1.409	0.119	1.528	1.291	1.461
**0.0010183**	0.875	0.875	0.032	0.907	0.843	0.148	**0.0027**	0.822	0.811	0.052	0.864	0.760	2.422
**0.010428**	1.317	1.307	0.102	1.41	1.206	1.508	**0.0015**	1.011	1.006	0.039	1.045	0.967	1.466
**0.0060534**	0.899	0.881	0.078	0.959	0.804	4.437							

**Table 4:** Statistical analysis outcome for the combination of parameters RR interval, JT interval, and QRS interval.

Healthy Candidates (H)							Unhealthy Candidates (UH)						
$\mathrm{Variance}\;\sigma_{H}^{2}$	$\mathrm{Mean}\;\mu_{H}$	$\mathrm{Median}\;x\sim_{H}$	$\mathrm{Standard}\;\mathrm{Deviation}\;\sigma_{H}$	$\mathrm{Upper}\;\mathrm{Limit}\;(x\sim_{UH}+\sigma_{UH})$	$\mathrm{Lower}\;\mathrm{Limit}\;(x\sim_{UH}-\sigma_{UH})$	$\mathrm{Area}\;A_{H}$	$\mathrm{Variance}\;\sigma_{UH}^{2}$	$\mathrm{Mean}\;\mu_{UH}$	$\mathrm{Median}\;x\sim_{UH}$	$\mathrm{Standard}\;\mathrm{Deviation}\;\sigma_{UH}$	$\mathrm{Upper}\;\mathrm{Limit}\;(x\sim_{UH}+\sigma_{UH})$	$\mathrm{Lower}\;\mathrm{Limit}\;(x\sim_{UH}-\sigma_{UH})$	$\mathrm{Area}\;A_{UH}$
**0.000792**	0.736	0.736	0.028	0.764	0.708	0.242	**0.0003**	1.168	1.164	0.018	1.182	1.146	0.775
**0.001677**	0.827	0.821	0.041	0.862	0.780	1.147	**0.0002**	1.282	1.279	0.015	1.294	1.264	0.896
**0.001713**	0.921	0.922	0.041	0.963	0.880	−0.494	**0.0061**	1.016	1.011	0.078	1.089	0.932	2.051
**0.000767**	1.033	1.033	0.026	1.061	1.005	0.310	**0.0006**	0.933	0.937	0.025	0.962	0.911	−1.264
**0.001689**	1.031	1.034	0.041	1.075	0.999	−1.086	**0.0141**	1.424	1.411	0.119	1.529	1.292	1.477
**0.001000**	0.881	0.879	0.032	0.911	0.848	0.231	**0.0027**	0.823	0.812	0.052	0.864	0.759	2.434
**0.01042**	1.319	1.310	0.102	1.412	1.208	1.525	**0.0015**	1.012	1.006	0.039	1.046	0.967	1.478
**0.006422**	0.906	0.891	0.080	0.976	0.810	4.392							

**Table 5:** Statistical analysis outcome for the combination of parameters JT interval, RR interval, and QRS interval.

Healthy Candidates (H)							Unhealthy Candidates (UH)						
$\mathrm{Variance}\;\sigma_{H}^{2}$	$\mathrm{Mean}\;\mu_{H}$	$\mathrm{Median}\;x\sim_{H}$	$\mathrm{Standard}\;\mathrm{Deviation}\;\sigma_{H}$	$\mathrm{Upper}\;\mathrm{Limit}\;(x\sim_{H}+\sigma_{H})$	$\mathrm{Lower}\;\mathrm{Limit}\;\left(x\sim_{H}-\sigma_{H}\right)$	$\mathrm{Area}\;A_{H}$	$\mathrm{Variance}\;\sigma_{UH}^{2}$	$\mathrm{Mean}\;\mu_{UH}$	$\mathrm{Median}\;x\sim_{UH}$	$\mathrm{Standard}\;\mathrm{Deviation}\;\sigma_{UH}$	$\mathrm{Upper}\;\mathrm{Limit}\;(x\sim_{UH}+\sigma_{UH})$	$\mathrm{Lower}\;\mathrm{Limit}\;(x\sim_{UH}-\sigma_{UH})$	$\mathrm{Area}\;A_{UH}$
**0.0013**	0.698	0.694	0.037	0.731	0.658	1.565	**0.0006**	1.397	1.393	0.025	1.418	1.367	0.9204
**0.0030**	0.882	0.880	0.055	0.934	0.825	0.527	**0.0004**	1.539	1.535	0.019	1.554	1.516	1.1994
**0.0025**	1.039	1.032	0.050	1.082	0.982	1.427	**0.0090**	1.051	1.043	0.095	1.138	0.948	2.2968
**0.0014**	1.165	1.168	0.037	1.206	1.131	−0.806	**0.0014**	1.070	1.077	0.037	1.114	1.040	−2.2801
**0.0043**	1.169	1.184	0.066	1.250	1.119	−5.280	**0.0067**	1.293	1.283	0.082	1.365	1.201	1.1756
**0.0029**	0.905	0.909	0.054	0.963	0.856	−0.469	**0.0031**	0.900	0.900	0.056	0.956	0.845	0.6442
**0.0031**	1.441	1.446	0.055	1.502	1.391	−1.105	**0.0014**	1.140	1.139	0.038	1.177	1.101	0.5454
**0.0021**	1.003	0.998	0.046	1.044	0.953	1.300							

**Table 6:** Statistical computations for the cohort of healthy and unhealthy individuals for the combination of parameters JT interval, QRS interval, and RR interval. The variance, mean, median, and standard deviation values are tabulated.

Healthy Candidates (H)							Unhealthy Candidates (UH)						
$\mathrm{Variance}\;\sigma_{H}^{2}$	$\mathrm{Mean}\;\mu_{H}$	$\mathrm{Median}\;x\sim_{H}$	$\mathrm{Standard}\;\mathrm{Deviation}\;\sigma_{H}$	$\mathrm{Upper}\;\mathrm{Limit}\;(x\sim_{H}+\sigma_{H})$	$\mathrm{Lower}\;\mathrm{Limit}\;\left(x\sim_{H}-\sigma_{H}\right)$	$\mathrm{Area}\;A_{H}$	$\mathrm{Variance}\;\sigma_{UH}^{2}$	$\mathrm{Mean}\;\mu_{UH}$	$\mathrm{Median}\;x\sim_{UH}$	$\mathrm{Standard}\;\mathrm{Deviation}\;\sigma_{UH}$	$\mathrm{Upper}\;\mathrm{Limit}\;(x\sim_{UH}+\sigma_{UH})$	$\mathrm{Lower}\;\mathrm{Limit}\;(x\sim_{UH}-\sigma_{UH})$	$\mathrm{Area}\;A_{UH}$
**0.0012**	0.692	0.689	0.035	0.724	0.654	1.370	**0.0006424**	1.397	1.393	0.025	1.418	1.367	0.904
**0.0030**	0.881	0.880	0.055	0.934	0.825	0.435	**0.0003502**	1.538	1.535	0.019	1.553	1.516	1.219
**0.0023**	1.032	1.025	0.048	1.073	0.978	1.565	**0.0082**	1.038	1.031	0.091	1.122	0.940	2.147
**0.0014**	1.161	1.164	0.037	1.201	1.127	−0.773	**0.0015**	1.065	1.073	0.039	1.112	1.034	−2.483
**0.0040**	1.162	1.176	0.064	1.240	1.113	−5.041	**0.0064**	1.285	1.274	0.080	1.354	1.194	1.532
**0.0025**	0.894	0.895	0.050	0.945	0.845	0.042	**0.0030**	0.897	0.900	0.055	0.955	0.844	0.355
**0.0031**	1.431	1.437	0.056	1.493	1.382	−1.224	**0.0014**	1.135	1.134	0.038	1.172	1.096	0.548
**0.0018**	0.991	0.987	0.042	1.029	0.945	1.159							

**Table 7:** Classification rules specified for the new incoming candidate into the variation interval.

	Condition 1	Condition 2	Condition 3
If	C $\leq \mu_{h}-\sigma_{h}$	$\;C\geq {\;\mu }_{\mathrm{uh}}+\sigma_{\mathrm{uh}}$	${\;\mu }_{\mathrm{uh}}-\sigma_{\mathrm{uh}}\leq C\leq {\;\mu }_{h}+\sigma_{h}$
Then, indicator is	IND=0	IND=1	$\mathrm{IND}=\frac{C-(\mu_{\mathrm{uh}}-\sigma_{\mathrm{uh}})}{\left(\mu_{h}+\sigma_{h}\right)-\left(\mu_{\mathrm{uh}}-\sigma_{\mathrm{uh}}\right)}$

## Data Availability

The data presented in this study are available on request from the corresponding author.
